# *FABP3* methylation as a novel biomarker for the differentiation and classification of benign and malignant thyroid nodules

**DOI:** 10.3389/fendo.2025.1630001

**Published:** 2025-09-11

**Authors:** Haixia Huang, Yifei Yin, Yizhu Mao, Hong Li, Junjie Li, Mengxia Li, Yi Zhang, Xuandong Huang, Yifen Zhang, Chenxia Jiang, Rongxi Yang

**Affiliations:** ^1^ Department of Epidemiology, School of Public Health, Nanjing Medical University, Nanjing, China; ^2^ Department of Thyroid and Breast Surgery, The Affiliated Huai’an Hospital of Xuzhou Medical University, The Second People’s Hospital of Huai’an, Huai’an, China; ^3^ Department of Pathology, The Affiliated Huai’an Hospital of Xuzhou Medical University, The Second People’s Hospital of Huai’an, Huai’an, China; ^4^ Jiangsu Province Hospital of Chinese Medicine, Affiliated Hospital of Nanjing University of Chinese Medicine, Nanjing, China; ^5^ Department of Pathology, The Affiliated Hospital of Nantong University, Nantong, China

**Keywords:** benign thyroid nodule, thyroid cancer, DNA methylation, *FABP3*, biomarker, classification

## Abstract

**Introduction:**

Differentiation between benign and malignant thyroid nodules has been a challenge in clinical practice. We aim to explore a novel biomarker to determine the malignancy of thyroid nodules.

**Methods:**

In the discovery study, 32 tissue samples from benign thyroid nodule (BTN) and thyroid cancer (TC) patients were analyzed by Methylation 850K array and RNA-Sequencing. TC associated *FABP3* methylation was further verified by mass spectrometry in two independent studies (221 BTN vs. 222 TC in Validation I and 191 BTN vs. 256 TC in Validation II). Logistic regression analysis and non-parametric tests were used for the analysis between groups.

**Results:**

Altered and inversely correlated methylation and expression in the *FABP3* gene in TC was found in the discovery study (*P* = 2.90E-05 for the methylation and *P* = 0.040 for the expression), and verified in the two validation studies (*P* values range from 0.012 to 6.30E - 10-12). *FABP3* methylation could sufficiently differentiate TC from BTN (AUC = 0.77), and could be further improved when combined with the BRAF^V600E^ mutations (AUC = 0.87). The association between *FABP3* hypomethylation and TC was enhanced in women, in patients with younger age, with larger tumor size and with lower FT3. *FABP3* methylation was varied in BTN and TC subtypes, with the highest level in adenoma and the lowest in anaplastic thyroid cancer.

**Conclusion:**

Our study suggested that altered *FABP3* methylation in tissue samples as a potential biomarker to distinguish malignant and benign thyroid nodules, and might be helpful for the pathological classification of TC.

## 1 Introduction

Thyroid cancer (TC) is the most common endocrine malignant tumors in adults, with an estimated total of more than 9.2 million new cases in 2020 worldwide (1). In China, the incidence of TC increased from 2.40/100,000 in 2003 to 13.75/100,000 in 2012, with an average annual increase of 20% (2, 3). In some provinces, such as Zhejiang Province, the incidence rate of TC ranks first among all cancers in women (4, 5). Differentiated thyroid cancer (DTC) has two subtypes, papillary thyroid cancer (PTC) and follicular thyroid cancer (FTC), which are the most common subtype of TC. The rarer subtypes are medullary thyroid cancer (MTC) and anaplastic thyroid cancer (ATC) (6–8). Thyroid nodules have a high incidence and a low malignant rate in the population. Although 60% of ultrasonography showed the presence of thyroid nodules, only 5% were eventually confirmed as malignant (9).

Accurate diagnosis of TC, including the judgment of benign and malignant thyroid nodules and classification of TC, is one of the current clinical challenges. Ultrasound-guided fine-needle aspiration biopsy (FNAB) remains crucial in the preoperative diagnosis of thyroid nodules (10). However, up to 15 - 30% of thyroid nodules evaluated by FNAB were cytologically indeterminate, which were usually diagnosed by postoperative pathological diagnosis (11). Ultrasound-guided FNAB, while clinically essential, faces critical limitations, including operator-dependent sampling errors that may miss malignant foci, frequent insufficient cellular material (occurring in ~20% of procedures), high infrastructure costs, requiring specialized cytopathology expertise, and prolonged turnaround times delaying clinical decisions (12, 13). These constraints reduce diagnostic accuracy for indeterminate nodules. Patients with uncertain nodules will be at risk of overdiagnosis or misdiagnosis. Therefore, reliable and practical biomarkers are urgently needed.

Determining the malignancy of thyroid nodules is becoming increasingly dependent on molecular pathological techniques (14), including genetic detection and epigenetic detection (15, 16). The role of BRAF, RAS, and RET gene mutations in TC has been widely recognized. BRAF mutation is known to be specific for PTC, and the reported positive rate ranges from 29 to 83% (17, 18). Because of the low sensitivity, compensating molecular diagnostic methods are needed (19). Afirma gene expression classifiers and ThyroSeq v3 are suitable for fresh aspirates and are expensive (20, 21). In addition, they are associated with a high rate of overdiagnosis due to low positive predictive value (22). These facts point to the need for a highly accurate diagnostic test for thyroid nodules using time-insensitive materials.

DNA methylation catalyzed by DNA methyltransferases (DNMTs) is one of the important epigenetic modifications controlling gene expression and maintaining genome structure. Alterations of DNA methylation are early molecular changes in human cancers and play an important role in tumorigenesis. As has been reported, the DNA methylation biomarkers offer early detection capability as epigenetic changes precede histopathological alterations, high specificity for tumor subtyping, and increasingly cost-effective analysis with standardized protocols (23). As a potential biomarker, changes in the methylation profile have been found in all types of cancer including TC (24). For example, Stephen et al. used quantitative methylation-specific polymerase chain reaction (PCR) to detect the promoter methylation status of 21 candidate genes on 329 formalin-fixed paraffin-embedded (FFPE), demonstrating that combined abnormal gene methylation helps clinically differentiate FTC and PTC from benign thyroid nodules (BTN) (25). Yim et al. evaluated DNA methylation in 109 thyroid specimens from BTN, PTCs and adjacent normal thyroid tissues using the Reduced Representation Bisulfite Sequencing (RRBS) and then performed validation in a retrospective cohort containing 65 thyroid nodules, suggesting epigenetic testing as a new molecular approach for thyroid diagnostics (26). Nevertheless, these studies were based on candidate approaches and were also limited by small sample size. So far, there are few screenings for the variant methylation signatures aiming to differentiate malignant and benign thyroid tumors, especially in large sample sizes.

To establish reliable methylation signatures overcoming FNAB’s technical barriers, this study utilized surgically resected FFPE tissues, which provide both definitive histopathological classification and sufficient DNA for the development of robust biomarkers. Here, we intended to discover and validate the abnormal DNA methylation alterations in TC compared to BTN in the Chinese population. Combining the genome-wide screening assay of Methylation 850K BeadChip array and RNA-Sequencing, we discovered TC associated differential methylation in the *FABP3* gene and performed further validations via mass spectrometry in case-control studies with a total of 890 patients from two clinical centers.

## 2 Materials and methods

### 2.1 Study design and population

The studies were approved by the Ethics Committee of the Nanjing Medical University. Informed consent was obtained from each recruited participant. In the discovery study, we collected 32 fresh-frozen tissue samples from 17 BTN subjects and 15 TC cases at the Affiliated Huai’an Hospital of Xuzhou Medical University from 2019 to 2020. Two independent studies were conducted on 890 FFPE tissue samples for validation. Validation Ⅰ: 222 TC patients and 221 age- and gender-matched BTN subjects were collected from the Affiliated Huai’an Hospital of Xuzhou Medical University and the Jiangsu Provincial Hospital of Chinese Medicine from 2013 to 2023. In the BTN group, 78.70% (174/221) were female, with a median (interquartile range, IQR) age of 53.00 (45.50 - 60.00) years; while in the TC group, 72.10% (160/222) were female, with a median (IQR) age of 50.00 (41.75 - 58.00) years. Validation Ⅱ: a total of 256 TC cases and 191 age- and gender-matched BTN subjects were collected from the Affiliated Hospital of Nantong University from 2017 to 2022. The median (IQR) age of BTN subjects and TC cases was 49.00 (35.00 - 55.00) and 50.00 (38.00 - 57.00) years old, and the proportions of females were 77.50% (148/191) and 77.30% (198/256), respectively. The detailed clinical characteristics of the participants are shown in **Table 1**, including tumor length, tumor size, lymph node involvement, tumor stage, thyroid-stimulating hormone (TSH) levels, free triiodothyronine (FT3) levels, free tetraiodothyronine acid (FT4) levels, and classification of BTN and TC.

**Table 1:** Clinical characteristics of the participants in two independent validations.

Variables	Group	Validation Ⅰ			Validation Ⅱ		
		BTN (n = 221)	TC (n = 222)	*P* value	BTN (n = 191)	TC (n = 256)	*P* value
Age (years)		53.00 (45.50-60.00)	50.00 (41.75-58.00)	**0.017**	49.00 (35.00-55.00)	50.00 (38.00-57.00)	0.531^*^
Gender	Female	174 (78.70%)	160 (72.10%)	0.104^$^	148 (77.50%)	198 (77.30%)	0.971^$^
	Male	47 (21.30%)	62 (27.90%)		43 (22.50%)	58 (22.70%)	
Tumor length (cm)		–	1.30 (0.90-2.00)		–	1.50 (1.20-2.00)	
Tumor size	T1	–	163 (73.40%)		–	161 (62.90%)	
	T2&3&4	–	56 (25.20%)		–	56 (21.90%)	
	Unknown	–	3 (1.40%)		–	39 (15.20%)	
Lymph node involvement	pN0	–	103 (46.40%)		–	73 (28.50%)	
	pN1	–	107 (48.20%)		–	130 (50.80%)	
	Unknown	–	12 (5.40%)		–	53 (20.70%)	
Tumor stage	Stage Ⅰ	–	172 (77.50%)		–	164 (64.10%)	
	Stage Ⅱ&III&IV	–	49 (22.10%)		–	39 (15.20%)	
	Unknown	–	1 (0.40%)		–	53 (20.70%)	
TSH (µIU/mL)		1.46 (0.89 - 2.36)	1.88 (1.20 - 3.15)	**0.001**	1.71 (1.09 - 2.52)	2.09 (1.45 - 2.92)	**0.002**
FT3 (pmol/L)		4.34 (3.60 - 4.84)	4.26 (3.67 - 4.82)	0.525^*^	4.93 (4.60 - 5.31)	4.88 (4.52 - 5.33)	0.663^*^
FT4 (pmol/L)		15.84 (13.00 - 18.33)	15.06 (10.92 - 17.52)	**0.029**	11.56 (10.52 - 12.66)	11.56 (10.46 - 12.65)	0.746^*^
Classification of BTN	adenoma	90 (40.72%)	–		106 (55.50%)	–	
	goiter	127 (57.47%)	–		68 (35.60%)	–	
	subacute thyroiditis	–	–		3 (1.57%)	–	
	lymphatic thyroiditis	4 (1.81%)	–		14 (7.33%)	–	
Classification of TC	PTC	–	171 (77.03%)		–	197 (76.95%)	
	FTC	–	19 (8.56%)		–	31 (12.11%)	
	MTC	–	27 (12.16%)		–	24 (9.38%)	
	ATC	–	5 (2.25%)		–	4 (1.56%)	

*P* values^*^ were calculated by the Mann-Whitney U test. *P* values^$^ were calculated by Chi-square test. Significant *P* values are in bold.

BTN, benign thyroid nodule; TC, thyroid cancer; TSH, thyroid stimulating hormone; FT3, free triiodothyronine; FT4, free tetraiodothyronine acid; PTC, papillary thyroid cancer; FTC, follicular thyroid cancer; MTC, medullary thyroid cancer; ATC, anaplastic thyroid cancer.

In the discovery study and two independent validations, the inclusion criteria for malignant nodules were as follows: (1) no distant metastases or other co-occurring cancers, (2) before any related treatment, and (3) complete clinical records. BTN patients were matched to TC cases by age, gender, and the year of diagnosis. Histopathological diagnosis was performed by two qualified pathologists in all cases, and the clinical TNM stage of each malignant case was determined according to the 8th edition American Joint Committee on Cancer Staging System (27).

### 2.2 EquationsIllumina methylation EPIC 850K BeadChip array and RNA-sequencing

**Figure 1:**
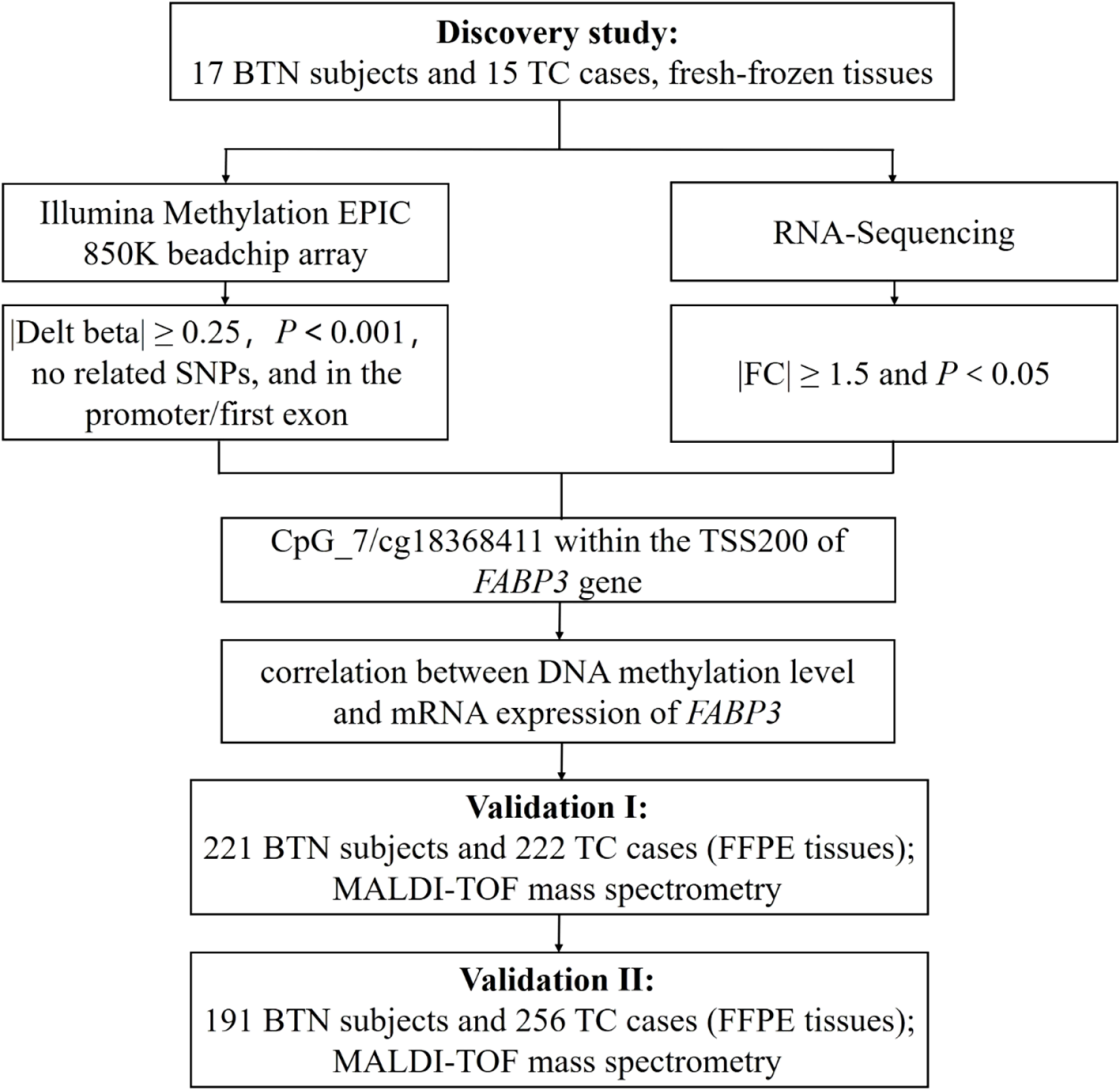
Study design and flow chart. The 32 fresh-frozen tissue samples in the discovery study were subjected to Illumina Methylation EPIC 850K BeadChip array and RNA-Sequencing. In the discovery study, there was a good correlation between DNA methylation and mRNA expression of *FABP3* in fresh-frozen tissue samples. Further validations with FFPE tissue samples were conducted in two independent studies (Validation Ⅰ and Validation Ⅱ) by MALDI-TOF mass spectrometry. BTN, benign thyroid nodule; TC, thyroid cancer; SNP, single nucleotide polymorphism; FC, fold change; TSS200, transcription start site 200 bp; FFPE, formalin-fixed and paraffin-embedded; MALDI-TOF, matrix-assisted laser desorption ionization time-of-flight.

The study design and flow chart were shown in **Figure 1**. In the discovery round, FastPure Blood/Cell/Tissue/Bacteria DNA Isolation Kit (DC112, Vazyme, Nanjing, China) and FastPure Cell/Tissue Total RNA Isolation Kit (RC101, Vazyme, Nanjing, China) were used to isolate genomic DNA and total RNA from 32 fresh-frozen tissue samples, respectively.

Genome-wide DNA methylation profiles were analyzed at single nucleotide resolution by the Illumina Methylation EPIC 850K BeadChip array. Probes that meet the following criteria were considered differentially methylated: (1) methylation difference between BTN and TC groups (|delta-beta|) ≥ 0.25, (2) *P* value < 0.001, (3) no adjacent single nucleotide polymorphisms (SNPs), and (4) on the promoter region or the 1^st^ exon of gene body. At the same time, mRNA expression was measured by RNA-Sequencing. Genes with a fold change of expression ≥ 1.5 and *P* value < 0.05 were thought to be differentially expressed.

### 2.3 Gene set enrichment analysis

GSEA was performed using the clusterProfiler R package (v4.4.4) on transcriptomic DEGs ranked by log2FoldChange. Gene IDs were converted to Entrez IDs via org.Hs.eg.db, with BH-adjusted *P* < 0.05. Key enriched pathways were visualized by enrichplot: gseaplot2 displaying enrichment score curves, gene positions, and ranking metrics. The tidyverse ecosystem was used for data processing.

### 2.4 Matrix-assisted laser desorption ionization time-of-flight mass spectrometry

DNA was extracted from FFPE tissue samples using FastPure FFPE DNA Isolation Kit (DC105, Vazyme, Nanjing, China). The isolated DNA was further bisulfite converted by EZ - 96 DNA Methylation Gold Kit (D5007, Zymo Research, Orange, USA) according to the manufacturer’s protocol. After bisulfite treatment, all non-methylated cytosine (C) bases in CpG sites were converted to uracil (U), whereas all methylated (C) bases remained unchanged. The CpG methylation levels of FFPE tissue samples were then determined by MALDI-TOF mass spectrometry, as described by Yang et al. and Yin et al. (28–30). Briefly, a 183 bp amplicon including 5 of the 7 significant *FABP3* CpG sites with adj.*P* < 0.05 from the 850K array (cg08877374, cg14407437, cg07345934, cg18368411 and cg15833534, **Supplementary Table S1**) was designed for analyses by MALDI-TOF mass spectrometry. The amplicons containing cg19316148 and cg20318096 failed in primer design. The target 183 bp amplicon was amplified by PCR using bisulfite-specific primers, forward primer: aggaagagagTTATAGTGATGTTGGGTTAGGTTGA, reverse primer: cagtaatacgactcactatagggagaaggctCAACCCCTCCTAAATAAACCCT. Upper case letters present the sequence-specific primer regions, and non-specific tags are shown in lower case letters. The sequence of the amplicon was presented in **Supplementary Table S2**, cg08877374 was referred to CpG_1, cg14407437 was referred to CpG_4, cg07345934 was referred to CpG_6, cg18368411 was referred to as CpG_7, and cg15833534 was referred to CpG_9. There are no SNPs overlapped with any of the CpG sites in the amplicon. Among the 11 CpG sites in this amplicon, the methylation levels of 7 sites were measurable by the Epityper assay including CpG_1/cg08877374, CpG_7/cg18368411 and CpG9/cg15833534, whereas the CpG_4/cg14407437 and CpG_6/cg07345934 were unmeasurable due to high mass. The 7 CpG sites yielded 5 distinguishable mass peaks by the mass spectrometry. CpG_9, CpG_10, and CpG_11 sites were located at the same fragment, and thus the mass peak shows the average methylation level of the three loci, which was presented as CpG_9.10.11. The other mass peaks have only one CpG locus. Next, the amplification products were treated with Shrimp Alkaline Phosphatase and followed by T-cleavage using RNase A. After cleaning residual ions with resin, the methylation level of each sample was quantified and collected by the MassARRAY system.

### 2.5 BRAF^V600E^ mutation detection

A single hotspot mutation in nucleotide 1799 of the BRAF gene (corresponding to p.V600E) has been recognized as the most frequent genetic event in PTC, with an incidence of 29 - 83%, and accounts for more than 90% of BRAF-mutated TC (31). A total of 711 patients from two validations were analyzed for BRAF gene mutations. Forward primer: 5’-TCATAATGCTTGCTGATAGGA-3’ and reverse primer: 5’-GGCCAAAAATTTAATCAGTGGA-3’ were used for amplification by PCR. The sequences of the amplified fragments were then analyzed by Sanger-Sequencing.

### 2.6 Statistical analyses

All data analyses were performed using SPSS (version 25.0) and GraphPad Prism (version 8.0). Mann-Whitney U test and Chi-square test were used to compare the differences between TC and BTN groups. Spearman correlation was used to determine the relationship between variables. Binary logistic regression analysis was performed to calculate odds ratios (ORs) and their 95% confidence intervals (95% CIs), adjusted for age, gender, TSH, FT3, and FT4 levels. In addition, subgroup analyses were stratified by gender adjusted for age, TSH, FT3, and FT4. Kruskal-Wallis test and Mann-Whitney U test were used to analyze the correlation between *FABP3* methylation levels and different clinical characteristics. Receiver operating characteristic (ROC) curve was used to evaluate goodness of fit. A two‐tailed *P* value < 0.05 was considered statistically significant.

## 3 Results

### 3.1 Discovery of TC associated *FABP3* hypomethylation in tissue

**Figure 2:**
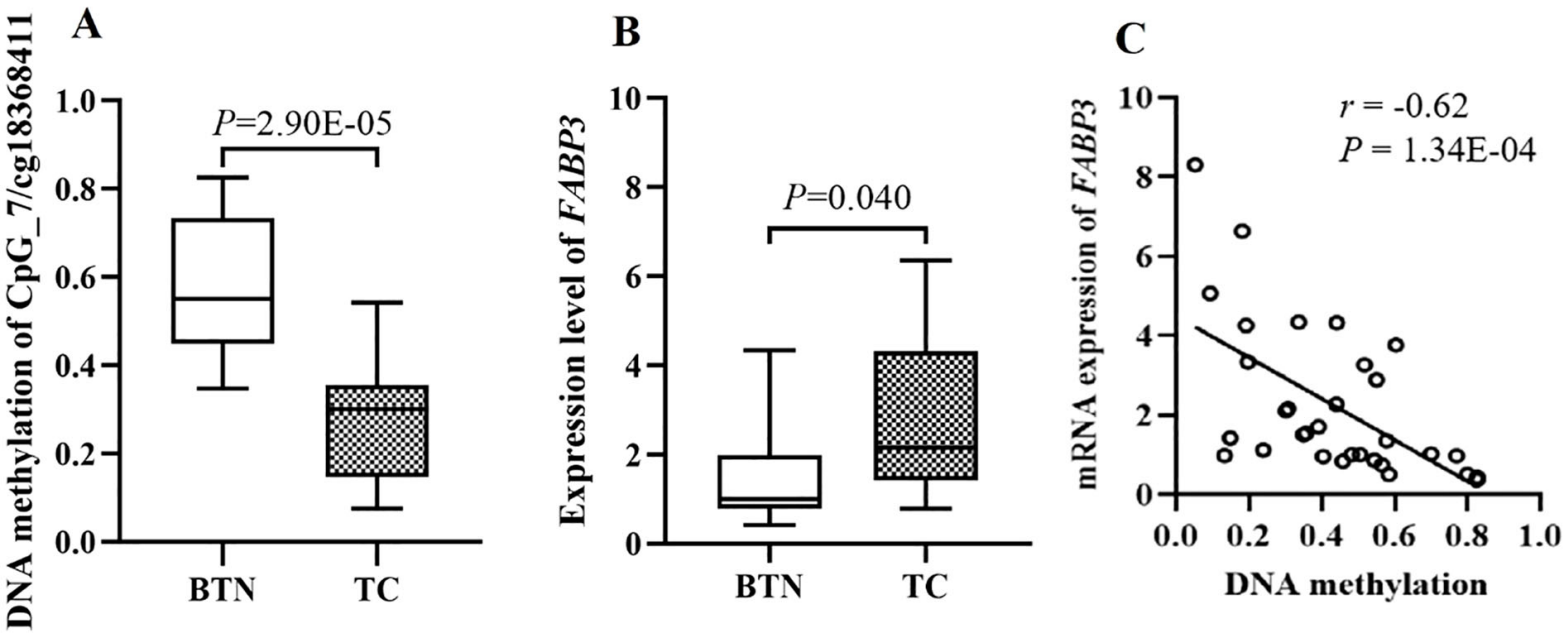
The discovery study showed significantly correlation between cg18368411 methylation and mRNA expression of the *FABP3* gene in fresh-frozen tissue samples. **(A)** Box plots for methylation levels of cg18368411 detected by Methylation 850K BeadChip array. **(B)** Box plots for mRNA expression levels of *FABP3* gene measured by RNA-Sequencing. **(C)** Correlations of *FABP3* expression with methylation levels of cg18368411.

Genome-wide Illumina Methylation 850K BeadChip array and RNA-Sequencing were used to screen methylation sites and genes with significant differences between TC and BTN in a total of 32 fresh-frozen samples (15 TC and 17 BTN). Gene Set Enrichment Analysis (GSEA) was applied to explore potential associations with pathways. As shown in **Supplementary Figure S1**, enrichment in fatty acid transport was observed. Fatty acid-binding protein 3 (*FABP3*), one of the central regulators of lipid metabolism and energy homeostasis, containing multiple TC-related aberrant methylation sites and also over-expressed in TC was considered for further investigation in our study. Cg18368411 within the transcription start site 200 bp (TSS200) of the *FABP3* gene (**Figure 1**) showed the most significant difference between TC cases and BTN subjects (median methylation: TC cases = 0.28, BTN subjects = 0.56, *P* = 2.90E-05; **Figure 2A**). Alterations in DNA methylation may affect gene expression. Consistently, we found that compared with BTN subjects, TC cases showed increased expression of *FABP3* mRNA (fold change =1.83, *P* = 0.040; **Figure 2B**). In addition, the methylation level of cg18368411 was significantly negatively correlated with the expression level of *FABP3* with the Spearman correlation coefficient value of -0.62 (*P* = 1.34E-04; **Figure 2C**). The Cancer Genome Atlas (TCGA)-THCA dataset (32–34) also supported our results that *FABP3* promoter methylation was marginally lower and the inversely correlated expression was higher in TC tissues compared to adjacent paired normal thyroid tissues (**Supplementary Figure S2**).

### 3.2 Validation of the differential *FABP3* methylation in BTN and TC by two independent case-control studies

**Figure 3:**
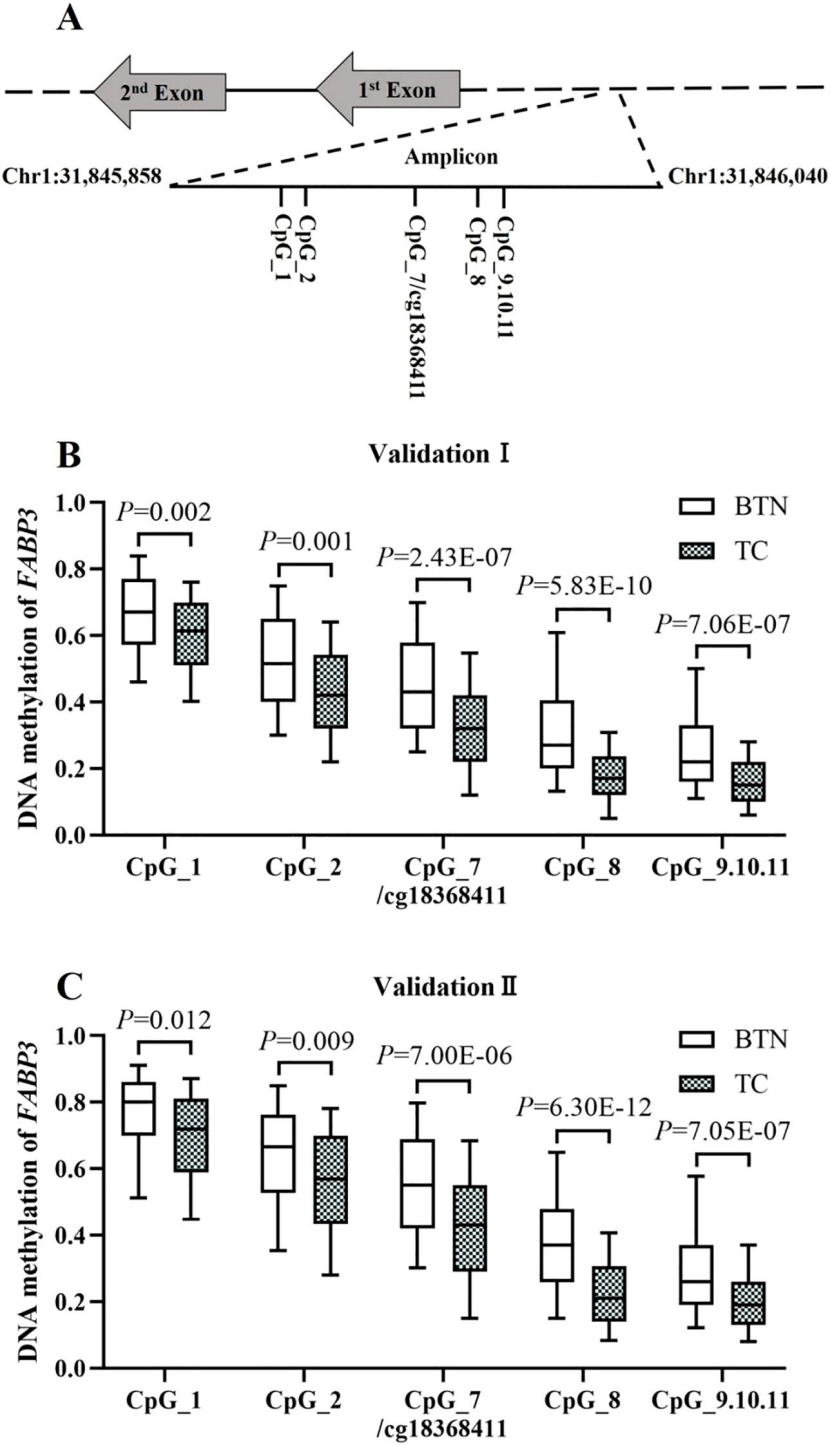
Validation of *FABP3* hypomethylation in TC cases compared to BTN subjects in two independent studies. **(A)** Schematic diagram of the target amplicon within the *FABP3* gene. A 183bp amplicon covers seven measurable CpG sites of the *FABP3* gene (Chr1: 31845858-31846040, build GRCh37/hg19, defined by the UCSC Genome Browser). Cg18368411 was referred to CpG_7. **(B, C)** Box plots for the methylation levels of the seven CpG sites in *FABP3* amplicon in Validation Ⅰ **(B)** and Validation Ⅱ **(C)**. All the *P* values were calculated by logistic regression adjusted for covariates. BTN, benign thyroid nodule; TC, thyroid cancer.

To validate *FABP3* hypomethylation in TC cases compared to BTN subjects, two independent case-control studies were conducted using FFPE tissue samples. A 183 bp amplicon containing cg18368411 site and flanking CpG sites was designed for analyses by MALDI-TOF mass spectrometry (**Figure 3A**).

In Validation I (222 TC cases and 221 age- and gender-matched BTN subjects), all the seven CpG sites in the *FABP3* amplicon showed significantly lower methylation levels in TC than in BTN, among which CpG_8 was the most significant loci (methylation values of BTN and TC: 0.27 vs. 0.17, *P* = 5.83E-10 adjusted for age, gender, TSH, FT3, and FT4). Moreover, there was a significant association between *FABP3* hypomethylation and TC cases. After adjusting for covariates, the odds ratios (ORs) per 10% reduced methylation of all *FABP3* CpG sites ranged from 1.24 to 1.81 (*P* ≤ 0.002 for all; **Figure 3B**, **Supplementary Table S3**). Consistent results were observed in Validation II (256 TC and 191 BTN). All seven *FABP3* CpG sites were hypomethylated in TC cases than those in BTN patients. Similarly, CpG_8 showed the most significant reduction (methylation values of BTN and TC: 0.37 vs. 0.21, *P* = 6.30E-12). Hypomethylation of all *FABP3* CpG sites was significantly associated with TC (the ORs per -10% methylation ranged from 1.17 to 1.79, *P* ≤ 0.012 for all by binary logistic regression adjusted for age, gender, TSH, FT3, and FT4; **Figure 3C**, **Supplementary Table S3**).

### 3.3 The association between *FABP3* hypomethylation in tissues and TC stratified by gender and age

To eliminate the confounding effects of gender and age, we further evaluated the association between *FABP3* methylation and TC cases by stratified regression analyses. To avoid possible bias due to the small sample size, the subjects in the two validations were combined (478 TC vs. 412 BTN).

**Figure 4:**
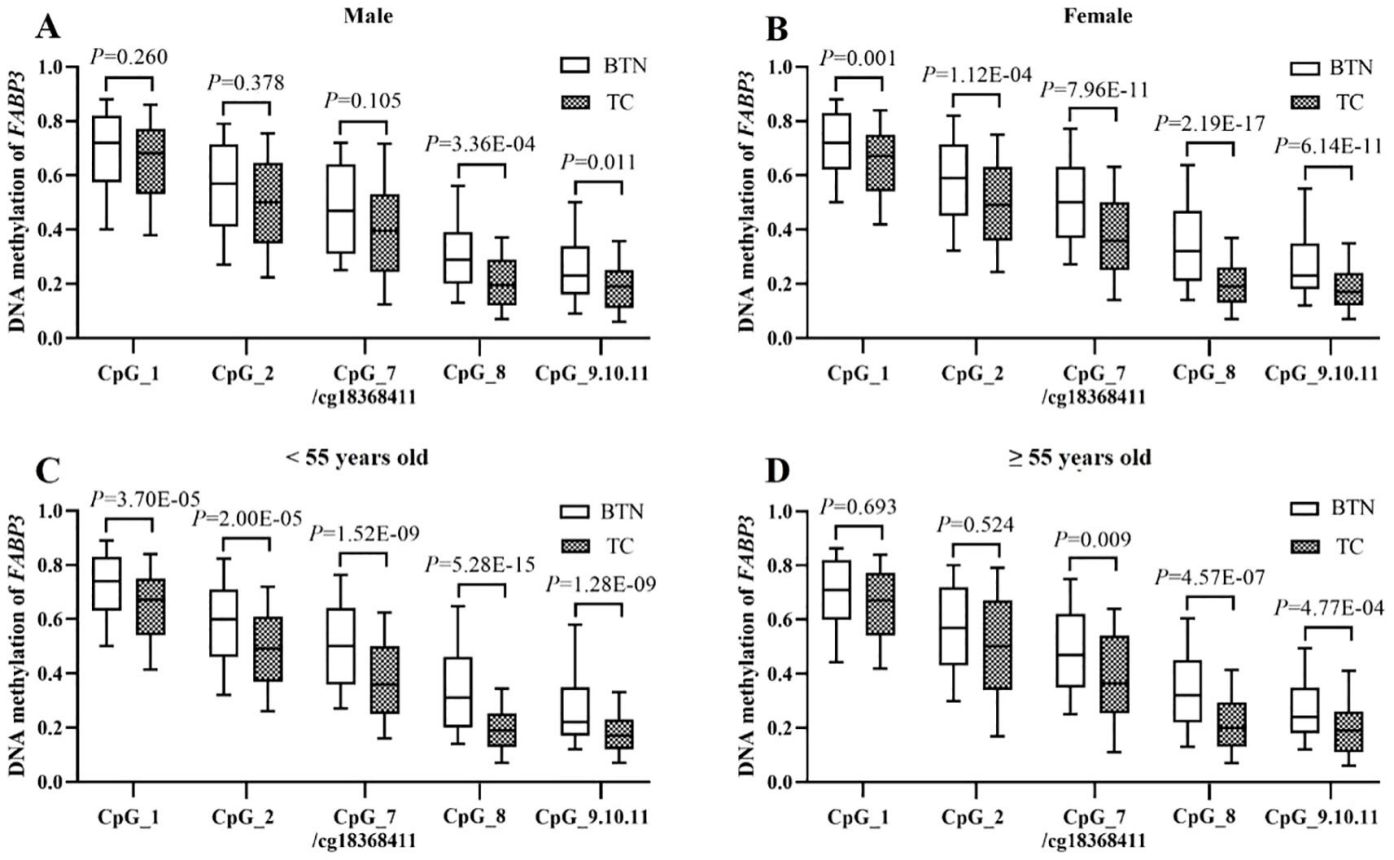
Combination analysis of the association between *FABP3* hypomethylation in FFPE tissues and TC stratified by age and gender. **(A, B)** Box plots for the *FABP3* methylation levels in the male group **(A)** and female group **(B)**. **(C, D)** Box plots for the *FABP3* methylation levels in the less than 55 years old group **(C)** and older than or equal to 55 years old group **(D)**. All the *P* values were calculated by logistic regression with the adjustment of covariates. BTN, benign thyroid nodule; TC, thyroid cancer.

When stratified by gender, in males, four out of seven *FABP3* CpG sites presented a significant association with TC (CpG_8 and CpG_9.10.11, the ORs per -10% methylation ranged from 1.39 to 1.56, all the *P* values ≤ 0.011; **Figure 4A**, **Supplementary Table S4**). In females, all CpG sites showed significantly lower methylation levels in TC cases than in BTN subjects (the ORs per -10% methylation ranged from 1.20 to 1.85, all the *P* values ≤ 0.001; **Figure 4B**, **Supplementary Table S4**) by logistic regression adjusted for age, TSH, FT3, and FT4. Moreover, the methylation differences between the cases and controls were larger in females than in males. When stratified by the age of 55 years old, which is the cutoff age for staging DTC, there was a significant association between all the seven CpG sites methylation and TC in the group less than 55 years old (the ORs per 10% reduced methylation ranged from 1.26 to 1.82, all the *P* values ≤ 3.70E - 05) by logistic regression adjusted for age, gender, TSH, FT3, and FT4 (**Figure 4C**, **Supplementary Table S5**). In contrast, in subjects older than or equal to 55 years old, only CpG_7/cg18368411, CpG_8, and CpG_9.10.11 sites showed association with TC (the ORs per -10% methylation ranged from 1.22 to 1.72, all the *P* values ≤ 0.009; **Figure 4D**, **Supplementary Table S5**).

### 3.4 The diagnostic efficiency of *FABP3* hypomethylation alone and combined with BRAF^V600E^ in differentiating TC from BTN

**Figure 5:**
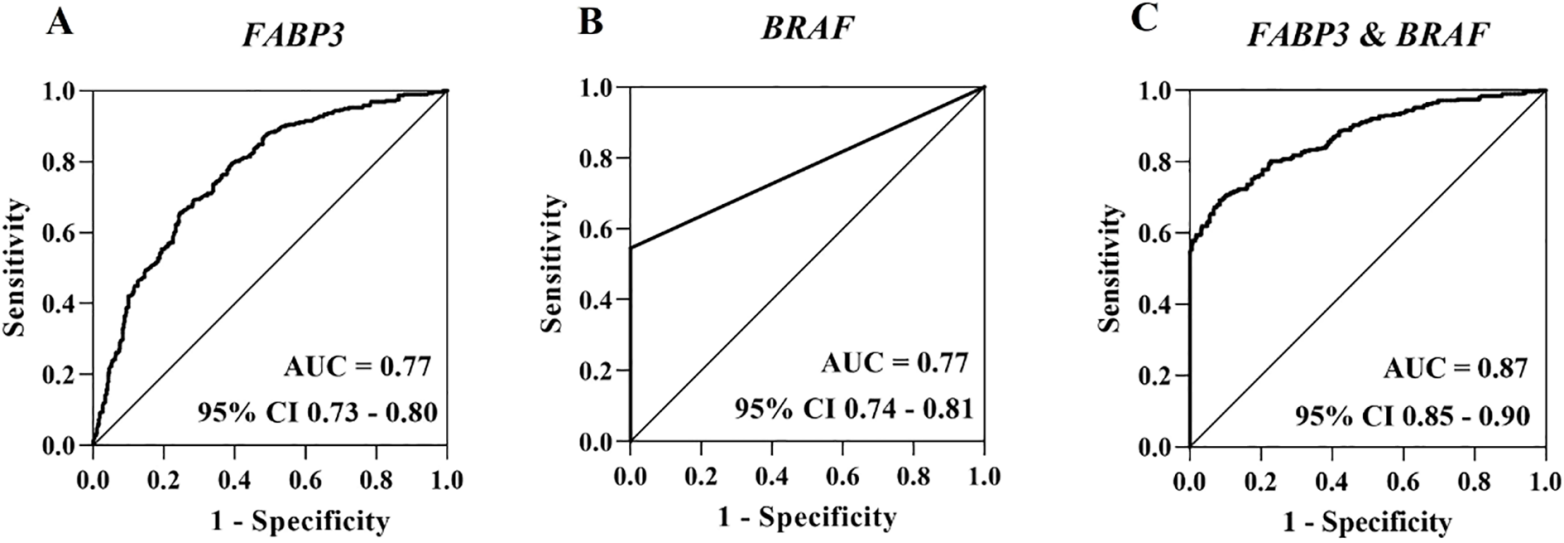
The diagnostic efficiency of *FABP3* hypomethylation alone and combined with BRAF^V600E^ in differentiating TC from BTN. **(A)** ROC curve analyses for the discriminatory power of the seven *FABP3* CpG sites to distinguish TC cases from BTN subjects. **(B)** ROC curve analysis for the discriminatory power of BRAF^V600E^ to distinguish TC cases from BTN subjects. **(C)** ROC curve analyses for the discriminatory power of *FABP3* methylation in combined with BRAF^V600E^ mutation to differentiate TC cases from BTN subjects. All the above 95% CI of AUC were calculated by logistic regression with covariates-adjusted. ROC, receiver operating characteristic; AUC, the area under the ROC curve; CI, confidence interval.

To estimate the potential clinical utility of *FABP3* methylation as a biomarker for TC, ROC curve analyses were performed and adjusted for age, gender, TSH, FT3, and FT4 by logistic regression. As shown in **Figures 5A**, *FABP3* hypomethylation based on FFPE tissue samples combining two validations has high credibility and accuracy in distinguishing TC cases from BTN patients (the area under the ROC curve (AUC) = 0.77, 95% CI: 0.73 - 0.80). The BRAF^V600E^ mutation has been reported to be a potential biomarker for PTC. Sanger-Sequencing was performed to evaluate the BRAF^V600E^ mutation status of the patients in two validations (**Supplementary Figure S3**). A total of 711 patients (381 BTN patients and 330 TC cases) were analyzed for mutations in the BRAF gene. No mutations were found in any of the BTN patients, while BRAF mutations were detected in 180 (54.5%) of the TC cases. We observed good predictability of BRAF^V600E^ to distinguish TC cases from BTN subjects (AUC = 0.77, 95% CI: 0.74 - 0.81; **Figure 5B**). Next, whether *FABP3* methylation in combination with BRAF^V600E^ could improve the diagnostic value of differentiating TC from BTN was investigated. The combined application of *FABP3* hypomethylation and BRAF^V600E^ achieved higher diagnostic accuracy (AUC = 0.87, 95% CI: 0.85 - 0.90; **Figure 5C**).

### 3.5 Histological classification of BTN and TC subtypes by *FABP3* methylation in tissue

**Figure 6:**
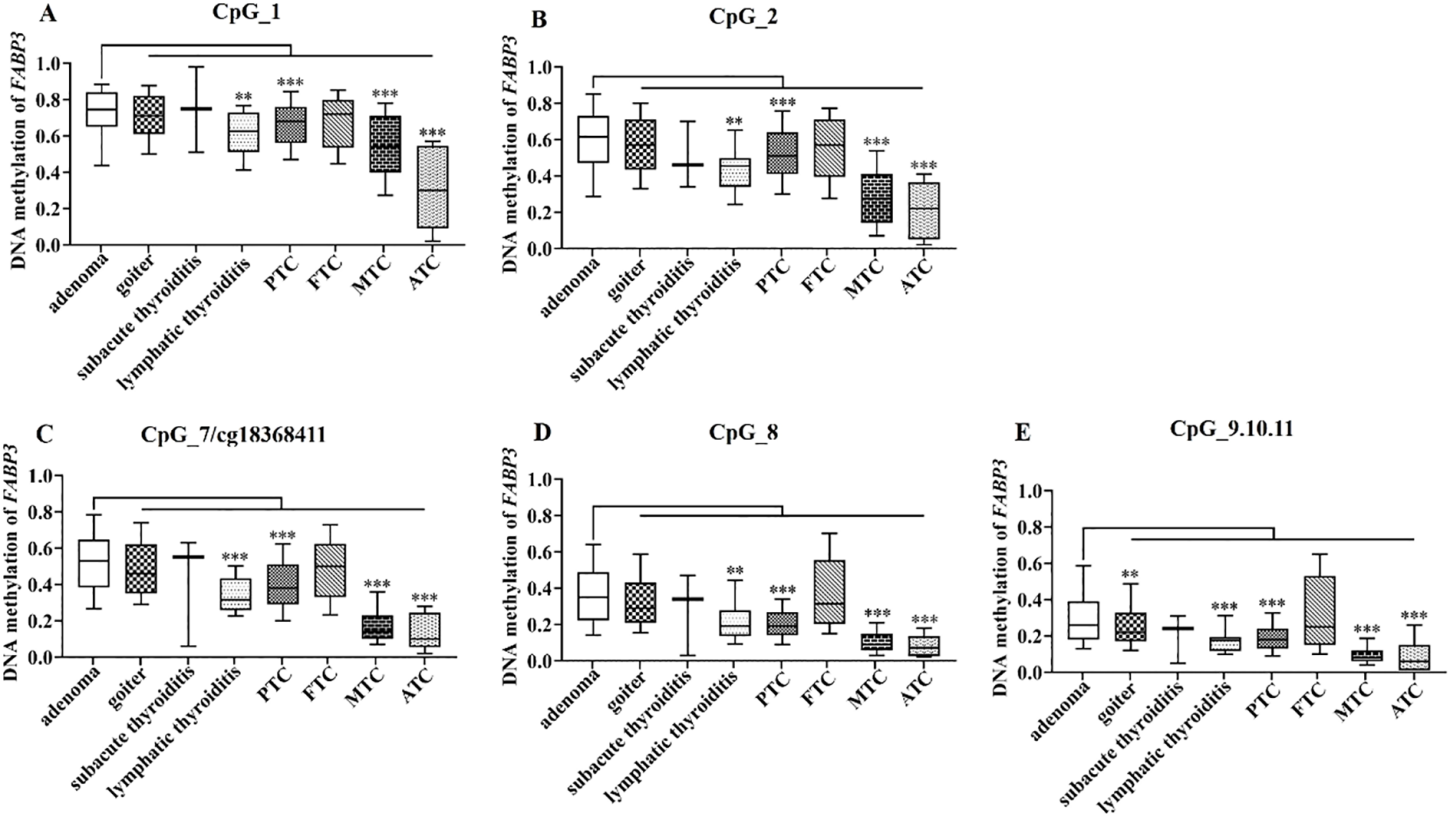
*FABP3* methylation in BTN and TC subtypes. **(A–E)** Box plots of methylation levels of seven CpG sites of *FABP3* in BTN and TC subtypes. All the *P* values were calculated by the Mann-Whitney U test referred to adenoma. *: *P* < 0.05, **: *P* < 0.01, ***: *P* < 0.001. BTN, benign thyroid nodule; TC, thyroid cancer; PTC, papillary thyroid cancer; FTC, follicular thyroid cancer; MTC, medullary thyroid cancer; ATC, anaplastic thyroid cancer.

Furthermore, we analyzed the methylation levels of *FABP3* in 478 TC cases and 412 BTN patients stratified by subtype. All seven *FABP3* CpG sites were hypomethylated in PTC, MTC, and ATC cases than those in adenoma patients (all the *P* values ≤ 5.90E - 05; **Figure 6**, **Supplementary Table S6**). However, we did not observe a difference in *FABP3* methylation between adenoma subjects and FTC cases (all the *P* values > 0.05; **Figure 6**, **Supplementary Table S6**). As for BTN, the methylation level of *FABP3* in patients with lymphatic thyroiditis was lower than that in patients with adenoma (all the *P* values ≤ 0.002; **Figure 6**, **Supplementary Table S6**). The most significant reduction was in CpG_7/cg18368411 (methylation values in adenoma and lymphatic thyroiditis: 0.53 vs. 0.32, *P* = 1.41E-04; **Figure 6**, **Supplementary Table S6**). In addition, we observed a correlation between five *FABP3* CpG sites methylation and tumor size (CpG_7/cg18368411, CpG_8, and CpG_9.10.11, all the *P* values ≤ 0.016; **Table 2**). Next, we evaluated the correlation between *FABP3* methylation and thyroid-related hormones with their median values as cutoff points (1.95 µIU/mL for TSH, 4.70 pmol/L for FT3, and 12.22 pmol/L for FT4). A positive correlation was observed between FT3 levels and the methylation levels of CpG_1/cg08877374, CpG_2, CpG_8, and CpG_9.10.11 sites (all the *P* values ≤ 0.046; **Table 2**).

**Table 2:** Correlation between *FABP3* methylation and the clinical characteristics of TC.

Clinical characteristics	Group (n)	Median of methylation levels				
		CpG_1/cg08877374	CpG_2	CpG_7/cg18368411	CpG_8	CpG_9.10.11
Tumor length (cm)	≤ 1.0 (126)	0.65 (0.55 - 0.72)	0.48 (0.37 - 0.58)	0.36 (0.29 - 0.46)	0.20 (0.15 - 0.25)	0.17 (0.13 - 0.22)
	1.0 - 2.0 (202)	0.68 (0.57 - 0.77)	0.51 (0.37 - 0.64)	0.38 (0.26 - 0.51)	0.19 (0.13 - 0.28)	0.18 (0.12 - 0.24)
	> 2.0 (94)	0.67 (0.48 - 0.81)	0.49 (0.28 - 0.67)	0.36 (0.17 - 0.54)	0.15 (0.09 - 0.28)	0.16 (0.07 - 0.27)
	*P* value^$^	0.087	0.226	0.641	0.136	0.481
	*FDR*	0.476	0.527	0.641	0.476	0.561
Tumor size	T1 (324)	0.67 (0.57 - 0.75)	0.50 (0.39 - 0.62)	0.37 (0.28 - 0.49)	0.20 (0.14 - 0.26)	0.18 (0.13 - 0.24)
	T2&3&4 (112)	0.62 (0.47 - 0.78)	0.45 (0.29 - 0.65)	0.30 (0.15 - 0.53)	0.15 (0.08 - 0.25)	0.15 (0.07 - 0.25)
	*P* value^*^	0.113	0.071	**0.006**	**0.001**	**0.016**
	*FDR*	0.113	0.083	**0.021**	**0.007**	**0.022**
Lymph node involvement	pN0 (176)	0.68 (0.55 - 0.76)	0.48 (0.37 - 0.61)	0.36 (0.25 - 0.50)	0.20 (0.14 - 0.30)	0.19 (0.12 - 0.25)
	pN1 (237)	0.67 (0.54 - 0.76)	0.51 (0.40 - 0.63)	0.36 (0.27 - 0.51)	0.19 (0.13 - 0.24)	0.17 (0.12 - 0.23)
	*P* value^*^	0.966	0.257	0.607	**0.033**	0.775
	*FDR*	0.966	0.900	0.904	0.231	0.904
Tumor stage	Stage Ⅰ (336)	0.67 (0.55 - 0.75)	0.50 (0.39 - 0.62)	0.37 (0.27 - 0.50)	0.19 (0.14 - 0.27)	0.18 (0.12 - 0.24)
	Stage Ⅱ&III&IV (88)	0.67 (0.51 - 0.78)	0.48 (0.33 - 0.63)	0.33 (0.16 - 0.53)	0.16 (0.09 - 0.25)	0.16 (0.07 - 0.24)
	*P* value^*^	0.524	0.199	0.050	**0.024**	0.076
	*FDR*	0.524	0.232	0.106	0.106	0.106
TSH (µIU/mL)	≤ 1.95 (183)	0.69 (0.58 - 0.78)	0.53 (0.41 - 0.65)	0.39 (0.30 - 0.51)	0.21 (0.14 - 0.28)	0.18 (0.13 - 0.25)
	> 1.95 (181)	0.68 (0.53 - 0.75)	0.50 (0.39 - 0.63)	0.37 (0.27 - 0.52)	0.19 (0.14 - 0.26)	0.18 (0.13 - 0.22)
	*P* value^*^	0.159	0.252	0.371	0.117	0.508
	*FDR*	0.508	0.508	0.508	0.508	0.508
FT3 (pmol/L)	≤ 4.70 (184)	0.67 (0.55 - 0.74)	0.47 (0.37 - 0.62)	0.36 (0.28 - 0.50)	0.18 (0.13 - 0.25)	0.17 (0.12 - 0.23)
	> 4.70 (180)	0.70 (0.57 - 0.79)	0.56 (0.43 - 0.65)	0.40 (0.31 - 0.53)	0.21 (0.15 - 0.30)	0.19 (0.13 - 0.26)
	*P* value^*^	**0.011**	**0.002**	0.060	**0.019**	**0.046**
	*FDR*	**0.039**	**0.014**	0.060	**0.044**	0.054
FT4 (pmol/L)	≤ 12.22 (183)	0.71 (0.58 - 0.79)	0.53 (0.41 - 0.67)	0.39 (0.30 - 0.55)	0.20 (0.14 - 0.28)	0.18 (0.13 - 0.24)
	> 12.22 (181)	0.66 (0.55 - 0.74)	0.50 (0.38 - 0.61)	0.37 (0.27 - 0.47)	0.19 (0.14 - 0.26)	0.18 (0.13 - 0.24)
	*P* value^*^	**0.011**	0.104	0.052	0.185	0.680
	*FDR*	0.077	0.243	0.182	0.324	0.680

*P* values^*^ were calculated by the Mann-Whitney U test. *P* values^$^ were calculated by the Kruskal-Wallis test. Significant *P* values are in bold.

TC, thyroid cancer; TSH, thyroid stimulating hormone; FT3, free triiodothyronine; FT4, free tetraiodothyronine acid; *FDR*, false discovery rate.

## 4 Discussion

Fatty acid-binding proteins (*FABPs*) are expressed in most major tissues (35), and have been proposed to be central regulators of lipid metabolism and energy homeostasis through their control of fatty acid transport (36). *FABP3*, which is mainly expressed in skeletal muscle, the heart, and the placenta, plays a role in the intracellular transport of long-chain fatty acids and their acyl-CoA esters (37). Recent reports have shown that *FABP3* may be involved in the pathogenesis of various diseases (38, 39). For example, knockdown of *FABP3* caused mitochondrial dysfunction and increased the apoptosis of cardiac cell lines (40). Bensaad et al. found that *FABP3* knockdown impaired the growth of glioblastoma xenograft by reducing fatty acid uptake and oxidation (41). The methylation of *FABP3* is associated with insulin, lipids, and cardiovascular phenotypes of the metabolic syndrome (42). As a fatty acid transporter, *FABP3* hypomethylation and consequently overexpression could enhance intracellular lipid flux and activate PPARγ/RXRα signaling (43), which is implicated in tumor proliferation of thyroid cancer (44). Till now, there have been no reports on *FABP3* methylation alterations in TC. Here, we discovered hypomethylation of the *FABP3* gene in TC cases compared to BTN subjects together with a markedly elevated *FABP3* mRNA expression via joint analyses of 850K BeadChip array and RNA-Sequencing, and performed further validations using mass spectrometry in two independent studies with large sample size.

Age and gender have been identified as risk factors for the incidence of TC (45). TC is the only malignancy with age as a prognostic indicator in the majority of staging systems (46), which is the most frequently diagnosed malignancy among adolescents and young adults (47, 48). Deng et al. reported that the most common onset age in persons who developed TC decreased, and the age at death of those with TC increased worldwide (49). A retrospective cohort evaluation of TC cases and deaths during 2005 – 2015 showed the rate of increasing incidence trend was higher in the younger age group and lower in the older age group (50). Our results revealed the association between altered *FABP3* methylation and TC risk, especially in younger people. Since global methylation levels decline with age (51), larger methylation differences could be expected among the younger individuals. As for gender, TC is the only non-reproductive cancer that occurs more often in women than in men, with a 3 - 4-fold higher incidence among women than men (52). We observed the methylation differences of the *FABP3* gene between the TC cases and BTN subjects were larger in females than in males. Estrogen may participate in the initiation of tumorigenesis in TC. It has been reported that estrogen has a positive effect on the proliferation of thyroid cells, which is essential for the development of TC, and may contribute to the mechanisms that cause DNA damage (53). In addition, estrogen has also been shown to enhance the secretion of VEGF (vascular endothelial growth factor) by thyroid cells (54, 55), thereby regulating the vascular environment and allowing further tumor growth (56, 57). Moreover, estrogen receptor plays a role in cell migration and invasion in TC. The observed stronger association between *FABP3* hypomethylation and malignancy in females may reflect hormonal related epigenetic influences. Biologically, *FABP3* operates within the PPAR signaling pathway (PPARγ/RXRα), which is modulated by sex hormones (58–60). Estrogen enhances PPARγ activity, potentially amplifying *FABP3*’s metabolic role in thyroid tissue and strengthening its methylation changes in females (61). While our study did not directly probe these mechanisms, future work should investigate hormonal regulation (e.g., ESR1/PPARγ crosstalk) and age-related *FABP3* epigenetic drift in thyroid carcinogenesis.

Not only for the differentiation of BTN and TC, we also found interesting variation of *FABP3* methylation in variant subtypes of BTN (adenoma, goiter, subacute thyroiditis, lymphatic thyroiditis) and TC (PTC, FTC, MTC, ATC). Adenoma showed the highest *FABP3* methylation among all the BTN and TC subgroups, followed by goiter and subacute thyroiditis. To our surprise, the methylation level of *FABP3* in most lymphocytic thyroiditis is similar to that of malignancy but not as other BTN subgroups. Chronic inflammation has been considered as a pro-tumor effect in TC (62, 63). Lymphocytic thyroiditis is the most common autoimmune disorder. Several studies have shown an epidemiological correlation between lymphocytic thyroiditis and TC, especially PTC (64, 65). Our study hereby supported that chronic inflammation may be a precancerous lesion or at least a highly correlated risk factor. However, we have to be aware that our finding is based on limited sample size of lymphocytic thyroiditis. Since most benign nodules especially the individuals with thyroiditis do not undergo surgery, we would call for enlarged studies based on multi-centers for further validation. In addition, we found that the methylated level of *FABP3* was correlated with the grade of malignancy. The methylation of *FABP3* gradually declined from low malignant PTC to the highly aggressive ATC. The methylation level of *FABP3* of MTC, the intermediate subtypes of TC, is around 50% lower than PTC, but higher than ATC. Follicular adenoma is the early stage of FTC with a slightly different DNA methylation pattern than that observed in the normal thyroid, implying a role in the initiation of malignancy (66). Sharing a common genetic background, follicular adenoma and FTC represents a significant diagnostic challenge at pre- and postsurgical differentiation (67). Our study showed similar *FABP3* methylation level in FTC with adenoma, indicating that follicular adenoma may share common epigenetic background with FTC as well (68). Taken together, the epigenetic alterations in TC subtypes could be helpful for the diagnosis and classification of tumors, and may even indicate the precancerous lesion and the malignant grade of TC.

Our current study intentionally focused on the diagnostic performance of *FABP3* methylation especially for early-stage cancer (stage I and II), and thus, in lack of the prognostic information while early-stage TC is often curable upon timely diagnosis and treatment. Nevertheless, we have observed lower levels of *FABP3* methylation in TC patients with larger tumors, suggesting that the decreasing methylation level of *FABP3* may also be associated with the proliferation of tumors. Previous study has shown that high expression of *FABPs* in gastric cancer is associated with disease progression, tumor aggressiveness, and poor patient survival (69). Further investigation in larger studies is necessary to validate the correlations between *FABP3* hypomethylation and TC progression as well as its prognostic value. Pan-cancer evidence suggests that while certain methylation markers can serve as standalone prognostic factors, robust prognostic power frequently emerges from integrated models combining multiple molecular features (70, 71). Future studies should evaluate *FABP3* methylation and other methylation markers in conjunction with established prognostic co-factors, such as specific microRNAs or immune-context markers (72, 73), to develop enhanced prognostic tools. Interestingly, we discovered that the methylation level of *FABP3* steadily rose as FT3 levels rose and fell as FT4 levels rose. The relationship between changed thyroid hormone levels and the risk of TC has been widely studied (74–76). Low FT3 and high FT4 concentrations are associated with shorter survival in patients with TC (77). Moreover, serum FT3 levels are negatively correlated with inflammation, which is also associated with TC (78). FT4 levels play different roles in different cancer types. In liver cancer, reduced FT4 levels indicate an increased risk of death; whereas in patients with primary breast cancer, elevated FT4 levels are associated with poor prognosis (76). To date, few studies have been conducted on the relationship between thyroid-related hormones and alterations in gene methylation (79). Thus, future prospective studies are warranted.

The present study is among the largest studies on differential diagnosis of TC and BTN with a total of 890 samples, which suggests hypomethylation of the *FABP3* gene might be biomarkers to differentiate benign and malignant thyroid tumors. Compared to the other established tissue-based markers for the diagnosis of TC (80–83), such as *GNB5* expression (AUC = 0.67), *PLIN3* expression (AUC = 0.81) and *EPHB2* (no difference between TC and normal thyroid tissue), *FABP3* methylation showed a sufficient diagnostic performance (AUC = 0.77). Moreover, the combination of *FABP3* methylation with existing diagnostic techniques, such as BRAF^V600E^ mutation, the diagnostic efficacy can be further improved (AUC = 0.87). The combination with additional TC-related mutations may further improve the diagnostic value of *FABP3* methylation. However, the TCGA dataset retrieved from cBioPortal shows that the occurrence of *RAS* mutations in only 6.9% of PTCs, while RET/PTC fusions are even rarer (1.88%) (32, 84, 85). Considering our current sample size, there is no sufficient statistical power to support combinatorial modeling with rare mutations. In addition, although our study based on Chinese population was in agreement with the TCGA database with samples from heterogenous genetic background, the possible bias on population heterogeneity could not be excluded. We look forward to the future international studies with multi-ethnic cohorts considering rare TC-related mutations for further exploration.

## 5 Conclusion

To sum up, this study revealed and proved hypomethylation of *FABP3* gene, together with a markedly elevated *FABP3* mRNA expression in TC cases compared to BTN subjects. *FABP3* showed significant methylation differences between TC and BTN, and among TC subtypes, suggesting the potential of DNA methylation as a novel pathological biomarker not only in discriminating malignant and BTN, but also in distinguishing between TC subtypes. Moreover, by combining *FABP3* methylation with BRAF^V600E^ mutation, the diagnostic efficacy can be significantly improved. Further investigation in a prospective multi-center study is needed before clinical practice.

## Data Availability

The original contributions presented in the study are included in the article/**Supplementary Material**. Further inquiries can be directed to the corresponding authors.
