# Supplementary material for: FABP3 methylation as a novel biomarker for the differentiation and classification of benign and malignant thyroid nodules
Source: Front Endocrinol (Lausanne). 2025 Sep 11;16:1630001. doi: 10.3389/fendo.2025.1630001 (PMC12462056; doi:10.3389/fendo.2025.1630001)
Supplement: Supplementary file 1 [file DataSheet1.docx]

Table S1. The primers and sequence of the *FABP3* amplicon

| Forward primer | aggaagagagTTATAGTGATGTTGGGTTAGGTTGA |
| --- | --- |
| Reverse primer | cagtaatacgactcactatagggagaaggctCAACCCCTCCTAAATAAACCCT |
| Sequence* | CCATAGTGATGCTGGGCTAGGCTGAGAGAAGCTACAAGAGAGCAGG**CG**TGCAAGGGCTC**CG**ACGGCGGCTCCCTGCCCGGGCTGCCGCTTTAAATAGCCCT**CG**CATCACATGAGGAGA**CG**TGGCCCA**CG**CCCC**CG**GCTCC**CG**AAATAGGAAGCCCCAGGCTAGGGCTCACCCAGGAGGGGCTG |

* Upper case letters indicated the sequence-specific primer regions and non-specific tags were shown in lower case letters. The EpiTyper assay detected the methylation levels of 7 CpG sites and yielded 5 distinguishable mass peaks. The CpG sites that could be measured are in bold. CpG sites annotated in the 850K array are underlined: cg08877374 refers to CpG_1, cg14407437 refers to CpG_4 (unmeasurable due to high mass), cg07345934 refers to CpG_6 (unmeasurable due to high mass), cg18368411 refers to CpG_7, and cg15833534 refers to CpG_9.

Table S2. Significant methylation sites in *FABP3* gene via 850K array

| *FABP3/*CpG sites | Chromosome | Hot spot region | Illumina 850K beadchip array | | | |
| --- | --- | --- | --- | --- | --- | --- |
|  |  |  | CpG methylation | | Δβ | adj.*P* |
|  |  |  | BTN | TC |  |  |
| **cg08877374** | chr1:31845904 | 5’UTR | 0.68 | 0.51 | -0.17 | 0.008 |
| **cg14407437** | chr1:31845923 | TSS200 | 0.62 | 0.48 | -0.14 | 0.027 |
| **cg07345934** | chr1:31845943 | TSS200 | 0.58 | 0.36 | -0.22 | 0.010 |
| **cg18368411** | chr1:31845959 | TSS200 | 0.56 | 0.28 | -0.28 | 0.002 |
| **cg15833534** | chr1:31845985 | TSS200 | 0.37 | 0.18 | -0.18 | 0.020 |
| cg19316148 | chr1:31846089 | TSS200 | 0.39 | 0.19 | -0.19 | 0.014 |
| cg20318096 | chr1:31846110 | TSS200 | 0.36 | 0.20 | -0.16 | 0.040 |

Note: UTR, untranslated region; TSS, transcription start site; Δβ, the difference in methylation levels; BTN, benign thyroid nodule; TC, thyroid cancer. The CpG sites contained in amplicon are shown in bold. Significant adj.*P* values are in bold.

Table S3. The association between *FABP3* methylation and TC in two independent validations

| Variates | BTN | TC | OR (95% CI) per -10% methylation^$^ | *P* value^$^ |
| --- | --- | --- | --- | --- |
|  | median (IQR) | median (IQR) |  |  |
| Validation Ⅰ (221 BTN subjects vs. 222 TC cases) | | | | |
| CpG_1/cg08877374 | 0.67 (0.57 - 0.77) | 0.62 (0.51 - 0.70) | 1.26 (1.09 - 1.45) | **0.002** |
| CpG_2 | 0.52 (0.40 - 0.65) | 0.42 (0.32 - 0.54) | 1.24 (1.09 - 1.41) | **0.001** |
| CpG_7/cg18368411 | 0.43 (0.32 - 0.58) | 0.32 (0.22 - 0.42) | 1.45 (1.26 - 1.67) | **2.43E-07** |
| CpG_8 | 0.27 (0.20 - 0.41) | 0.17 (0.12 - 0.24) | 1.81 (1.50 - 2.19) | **5.83E-10** |
| CpG_9.10.11 | 0.22 (0.16 - 0.33) | 0.15 (0.10 - 0.22) | 1.67 (1.36 - 2.04) | **7.06E-07** |
| Validation Ⅱ (191 BTN subjects vs. 256 TC cases) | | | | |
| CpG_1/cg08877374 | 0.80 (0.70 - 0.86) | 0.72 (0.59 - 0.81) | 1.18 (1.04 - 1.35) | **0.012** |
| CpG_2 | 0.67 (0.53 - 0.76) | 0.57 (0.43 - 0.70) | 1.17 (1.04 - 1.32) | **0.009** |
| CpG_7/cg18368411 | 0.55 (0.42 - 0.69) | 0.43 (0.29 - 0.55) | 1.31 (1.16 - 1.47) | **7.00E-06** |
| CpG_8 | 0.37 (0.26 - 0.48) | 0.21 (0.14 - 0.31) | 1.79 (1.51 - 2.11) | **6.30E-12** |
| CpG_9.10.11 | 0.26 (0.19 - 0.37) | 0.19 (0.13 - 0.26) | 1.52 (1.29 - 1.80) | **7.05E-07** |

^$^Logistic regression adjusted for age, gender, TSH, FT3, and FT4. Significant *P* values are in bold.

Abbreviation: BTN, benign thyroid nodule; TC, thyroid cancer; IQR, interquartile range; CI, confidence interval; TSH, thyroid stimulating hormone; FT3, free triiodothyronine; FT4, free tetraiodothyronine acid.

Table S4. The association between *FABP3* methylation and TC stratified by gender combining Validation I and Validation II

| Variates | BTN | TC | OR (95% CI) per -10% methylation^$^ | *P* value^$^ |
| --- | --- | --- | --- | --- |
|  | median (IQR) | median (IQR) |  |  |
| male | (n = 90) | (n = 120) |  |  |
| CpG_1/cg08877374 | 0.72 (0.58 - 0.82) | 0.68 (0.53 - 0.77) | 1.11 (0.93 - 1.31) | 0.260 |
| CpG_2 | 0.57 (0.41 - 0.72) | 0.50 (0.35 - 0.65) | 1.08 (0.91 - 1.28) | 0.378 |
| CpG_7/cg18368411 | 0.47 (0.31 - 0.64) | 0.40 (0.24 - 0.53) | 1.15 (0.97 - 1.36) | 0.105 |
| CpG_8 | 0.29 (0.20 - 0.39) | 0.20 (0.12 - 0.29) | 1.56 (1.22 - 1.98) | **3.36E-04** |
| CpG_9.10.11 | 0.23 (0.16 - 0.34) | 0.19 (0.11 - 0.25) | 1.39 (1.08 - 1.78) | **0.011** |
| female | (n = 322) | (n = 358) |  |  |
| CpG_1/cg08877374 | 0.72 (0.62 - 0.83) | 0.67 (0.54 - 0.75) | 1.21 (1.09 - 1.36) | **0.001** |
| CpG_2 | 0.59 (0.45 - 0.72) | 0.49 (0.36 - 0.63) | 1.20 (1.10 - 1.32) | **1.12E-04** |
| CpG_7/cg18368411 | 0.50 (0.37 - 0.63) | 0.36 (0.25 - 0.50) | 1.41 (1.27 - 1.56) | **7.96E-11** |
| CpG_8 | 0.32 (0.21 - 0.47) | 0.19 (0.13 - 0.26) | 1.85 (1.61 - 2.14) | **2.19E-17** |
| CpG_9.10.11 | 0.23 (0.18 - 0.35) | 0.17 (0.12 - 0.24) | 1.66 (1.43 - 1.93) | **6.14E-11** |

^$^Logistic regression adjusted for age, TSH, FT3, and FT4. Significant *P* values are in bold.

Abbreviation: BTN, benign thyroid nodule; TC, thyroid cancer; IQR, interquartile range; CI, confidence interval; TSH, thyroid stimulating hormone; FT3, free triiodothyronine; FT4, free tetraiodothyronine acid.

Table S5. The association between *FABP3* methylation and TC stratified by age combining Validation I and Validation II

| Variates | BTN | TC | OR (95% CI) per -10% methylation^$^ | *P* value^$^ |
| --- | --- | --- | --- | --- |
|  | median (IQR) | median (IQR) |  |  |
| < 55 years old | (n = 257) | (n = 316) |  |  |
| CpG_1/cg08877374 | 0.74 (0.63 - 0.83) | 0.67 (0.54 - 0.75) | 1.30 (1.15 - 1.47) | **3.70E-05** |
| CpG_2 | 0.60 (0.46 - 0.71) | 0.49 (0.37 - 0.61) | 1.26 (1.13 - 1.40) | **2.00E-05** |
| CpG_7/cg18368411 | 0.50 (0.36 - 0.64) | 0.36 (0.25 - 0.50) | 1.39 (1.25 - 1.54) | **1.52E-09** |
| CpG_8 | 0.31 (0.20 - 0.46) | 0.19 (0.13 - 0.25) | 1.82 (1.57 - 2.11) | **5.28E-15** |
| CpG_9.10.11 | 0.22 (0.17 - 0.35) | 0.17 (0.12 - 0.23) | 1.63 (1.39 - 1.90) | **1.28E-09** |
| ≥ 55 years old | (n = 155) | (n = 161) |  |  |
| CpG_1/cg08877374 | 0.71 (0.60 - 0.82) | 0.67 (0.54 - 0.77) | 1.03 (0.89 - 1.20) | 0.693 |
| CpG_2 | 0.57 (0.43 - 0.72) | 0.50 (0.34 - 0.67) | 1.04 (0.92 - 1.19) | 0.524 |
| CpG_7/cg18368411 | 0.47 (0.35 - 0.62) | 0.37 (0.25 - 0.54) | 1.22 (1.05 - 1.41) | **0.009** |
| CpG_8 | 0.32 (0.22 - 0.45) | 0.20 (0.13 - 0.30) | 1.72 (1.39 - 2.12) | **4.57E-07** |
| CpG_9.10.11 | 0.24 (0.18 - 0.35) | 0.19 (0.11 - 0.26) | 1.49 (1.19 - 1.86) | **4.77E-04** |

^$^Logistic regression adjusted for age, gender, TSH, FT3, and FT4. Significant *P* values are in bold.

Abbreviation: BTN, benign thyroid nodule; TC, thyroid cancer; IQR, interquartile range; CI, confidence interval; TSH, thyroid stimulating hormone; FT3, free triiodothyronine; FT4, free tetraiodothyronine acid.

Table S6. *FABP3* methylation in BTN and TC subtypes

| Clinical characteristics | Group (n) | median of methylation levels | | | | |
| --- | --- | --- | --- | --- | --- | --- |
|  |  | CpG_1/cg08877374 | CpG_2 | CpG_7/cg18368411 | CpG_8 | CpG_9.10.11 |
| Classification of BTN | adenoma (196) | 0.75 (0.65 - 0.84) | 0.62 (0.47 - 0.73) | 0.53 (0.38 - 0.65) | 0.35 (0.22 - 0.49) | 0.26 (0.18 - 0.39) |
|  | goiter (195) | 0.71 (0.61 - 0.82) | 0.57 (0.44 - 0.71) | 0.46 (0.35 - 0.62) | 0.29 (0.21 - 0.43) | 0.22 (0.17 - 0.33) |
|  | subacute thyroiditis (3) | 0.75 (0.51 - 0.98) | 0.46 (0.34 - 0.70) | 0.55 (0.06 - 0.63) | 0.34 (0.03 - 0.47) | 0.24 (0.05 - 0.31) |
|  | lymphatic thyroiditis (18) | 0.63 (0.51 - 0.73) | 0.46 (0.34 - 0.50) | 0.32 (0.26 - 0.43) | 0.19 (0.14 - 0.28) | 0.18 (0.12 - 0.19) |
|  | *P* value^a^ | 0.111 | 0.245 | 0.061 | 0.058 | **0.008** |
|  | *P* value^b^ | 0.793 | 0.382 | 0.646 | 0.582 | 0.407 |
|  | *P* value^c^ | **0.002** | **0.001** | **1.41E-04** | **0.001** | **2.02E-04** |
| Classification of TC | PTC (368) | 0.68 (0.56 - 0.76) | 0.51 (0.41 - 0.64) | 0.38 (0.29 - 0.51) | 0.19 (0.14 - 0.27) | 0.18 (0.13 - 0.24) |
|  | FTC (50) | 0.72 (0.54 - 0.80) | 0.57 (0.40 - 0.71) | 0.50 (0.33 - 0.62) | 0.32 (0.20 - 0.56) | 0.25 (0.15 - 0.53) |
|  | MTC (51) | 0.54 (0.40 - 0.71) | 0.28 (0.14 - 0.41) | 0.14 (0.10 - 0.23) | 0.09 (0.06 - 0.15) | 0.08 (0.06 - 0.12) |
|  | ATC (9) | 0.30 (0.09 - 0.55) | 0.22 (0.05 - 0.37) | 0.10(0.06-0.25) | 0.07(0.03-0.14) | 0.06 (0.01 - 0.15) |
|  | *P* value^d^ | **2.00E-06** | **4.00E-06** | **3.04E-13** | **5.51E-24** | **1.14E-15** |
|  | *P* value^e^ | 0.095 | 0.232 | 0.275 | 0.659 | 0.782 |
|  | *P* value^f^ | **1.63E-08** | **2.97E-15** | **2.76E-19** | **1.27E-18** | **4.56E-19** |
|  | *P* value^g^ | **1.40E-05** | **1.80E-05** | **3.00E-06** | **7.00E-06** | **5.90E-05** |

All the *P* values were calculated by the Mann-Whitney U test. Significant P values are in bold.

*P* value^a^: adenoma vs. goiter; *P* value^b^: adenoma vs. subacute thyroiditis; *P* value^c^: adenoma vs. lymphatic thyroiditis; *P* value^d^: adenoma vs. PTC; *P* value^e^: adenoma vs. FTC; *P* value^f^: adenoma vs. MTC; *P* value^g^: adenoma vs. ATC.

Abbreviation: BTN, benign thyroid nodule; TC, thyroid cancer; PTC, papillary thyroid cancer; FTC, follicular thyroid cancer; MTC, medullary thyroid cancer; ATC, anaplastic thyroid cancer.


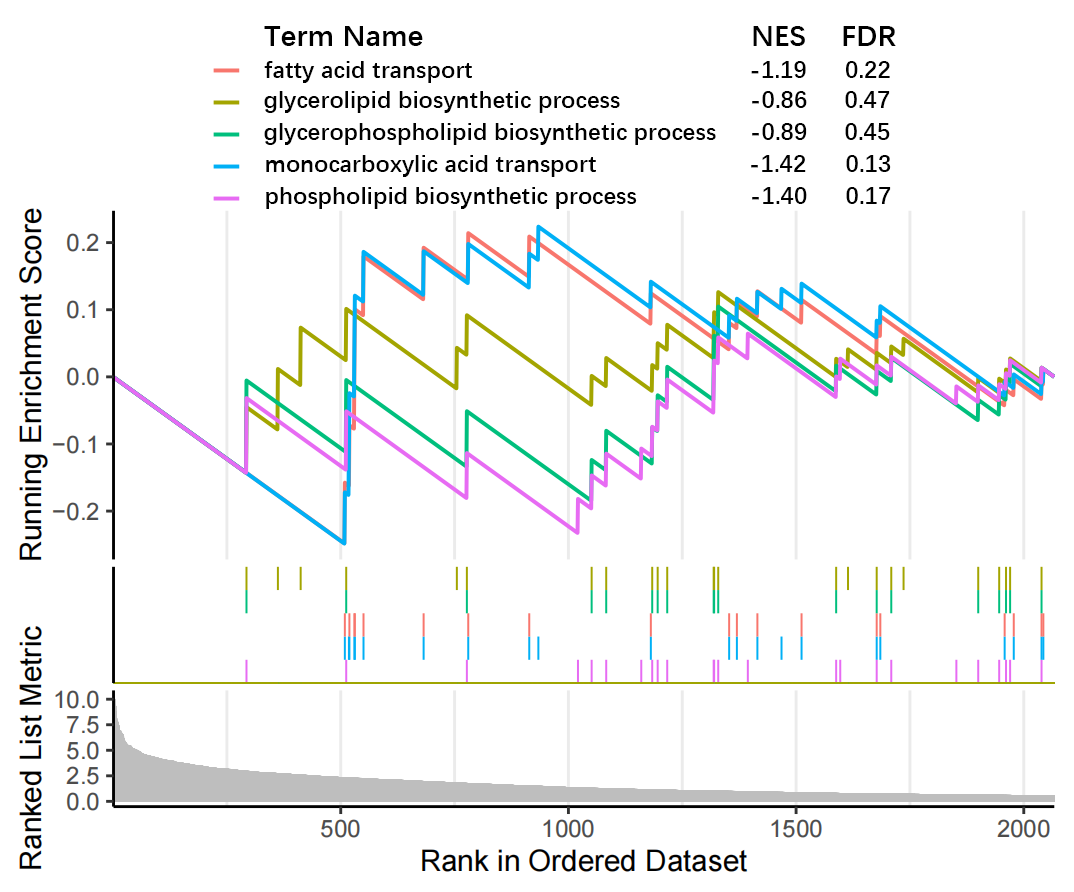


Figure S1. Gene Set Enrichment Analysis (GSEA) of *FABP3*-Associated Lipid Metabolic Pathways based on RNA-seq results of 17 BTN samples and 15 TC. The x-axis (Rank in Ordered Dataset) represents gene expression ranked by log2 fold-change from the most to the least significant; the y-axis shows the running enrichment score (ES), indicating the concentration of pathway genes in the ranked list. Genes in each pathway are marked by colored vertical ticks above the x-axis. The gray bar plot at the bottom displays the ranking metric. |Normalized enrichment score (NES) | > 1.0 and FDR < 0.25 was applied to indicated the significance. Colored curves depict ES profiles for: Red: Fatty acid transport (NES = -1.19, FDR = 0.22); Orange: Glycerolipid biosynthetic process (NES = -0.86, FDR = 0.47); Cyan: Glycerophospholipid biosynthetic process (NES = -0.89, FDR = 0.45); Blue: Monocarboxylic acid transport (NES = -1.42, FDR = 0.13); Purple: Phospholipid biosynthetic process (NES = -1.40, FDR = 0.17).


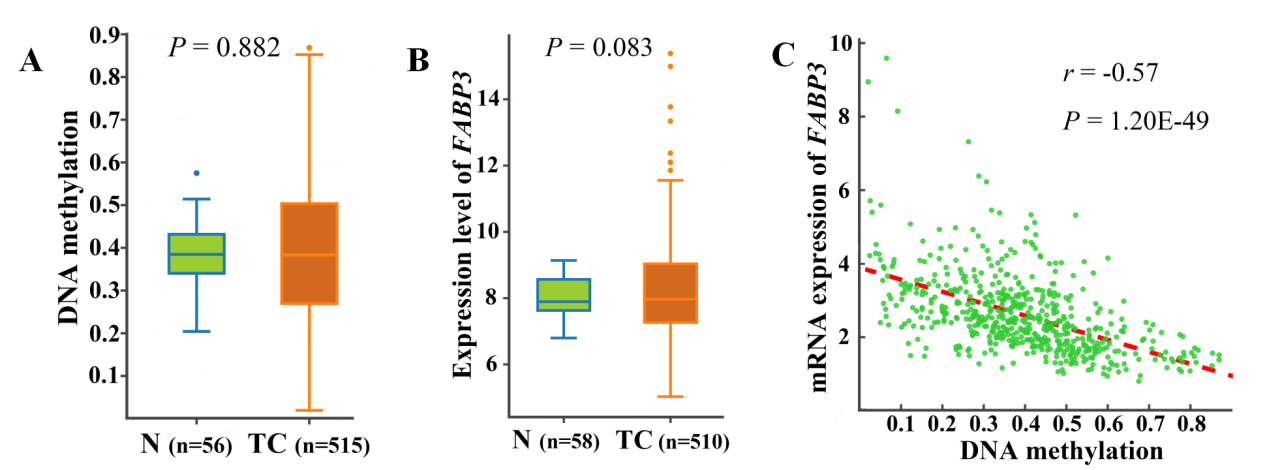


Figure S2. Significant correlation between *FABP3* methylation and mRNA expression in TCGA-THCA as revealed by the DNA Methylation Interactive Visual Database (DNMIVD). (A) Differential methylation levels of *FABP3* in the promoter region between TC and paired adjacent normal thyroid tissue (N). (B) Differential *FABP3* expression comparing TC with paired adjacent normal tissue (N). (C) Correlations between *FABP3* methylation and expression.


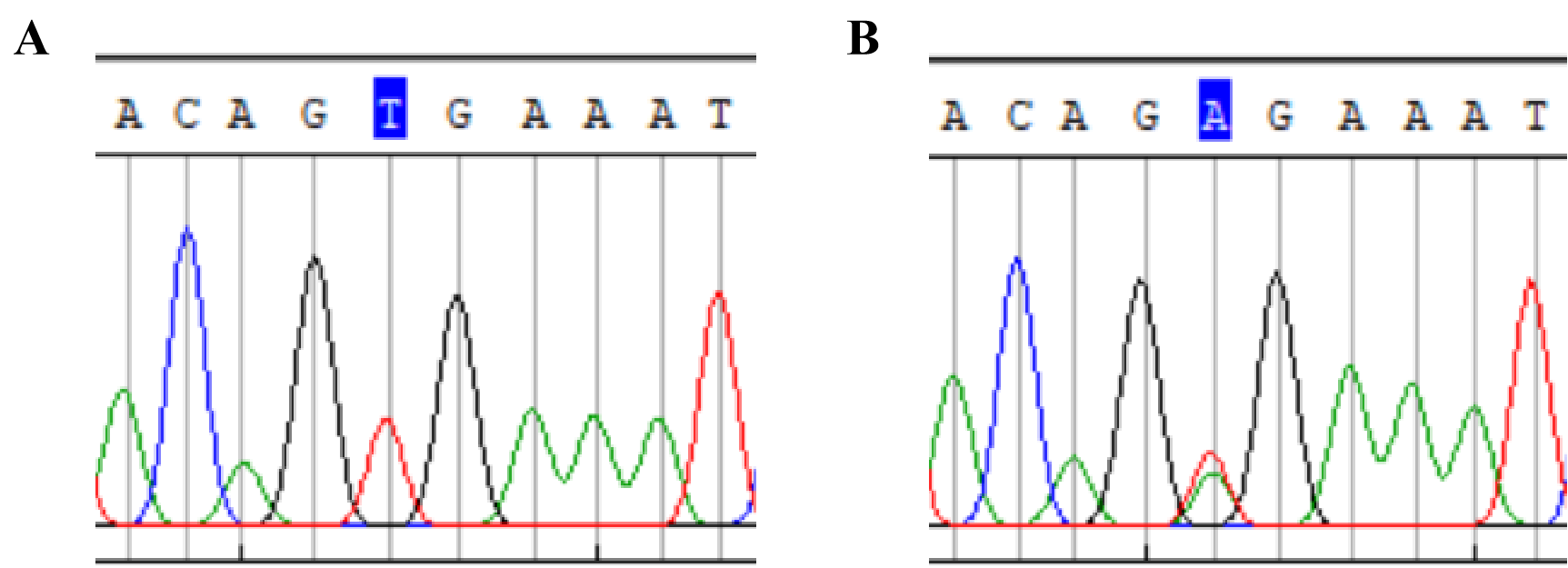


Figure S3. Representative Sanger-sequencing chromatogram. An amplicon covering the nucleotide 1799 of the BRAF gene (marked in blue, corresponding to p.V600E) was sequenced in all the 711 thyroid tumor tissues. (A) The representative sequencing chromatogram of homozygote wildtype 1799TT. (B) The representative sequencing chromatogram of heterozygote 1799TA, indicating the existence of the T>A non-synonymous mutation (V600E mutation).
